# *SpHMA3*: A Genetic Boost for Cadmium Tolerance and Bioremediation in *Arabidopsis thaliana* and *Zea mays*

**DOI:** 10.3390/ijms26083487

**Published:** 2025-04-08

**Authors:** Rumin Pu, Gaojiao Hu, Qian Jiang, Wenhao Zhou, Binhan Zhao, Chao Xia, Jianfeng Hu, Wenqi Xiang, Mao Liu, Hanyu Deng, Shuang Zhao, Jialong Han, Guihua Lv, Haijian Lin

**Affiliations:** 1 State Key Laboratory of Crop Gene Exploration and Utilization in Southwest China, Maize Research Institute, Sichuan Agricultural University, Chengdu 611130, China; prm20000401@163.com (R.P.); 18884770258@163.com (G.H.); jq20210707@163.com (Q.J.); zwh12030709@163.com (W.Z.); 15181787339@163.com (B.Z.); chaoxia@sicau.edu.cn (C.X.); hif14578760@163.com (J.H.); xwq150123@163.com (W.X.); 19162803124@163.com (M.L.); 17386742001@163.com (H.D.); 18384314783@163.com (S.Z.); hjl99819981@163.com (J.H.); 2 Institute of Maize and Featured Upland Crops, Zhejiang Academy of Agricultural Sciences, Dongyang 322100, China

**Keywords:** *maize*, cadmium, *SpHMA3*, functional analysis

## Abstract

In China, soil contamination by heavy metals is a widespread issue, with substantial increases in lead(Pb), cadmium(Cd), copper(Cu), and zinc(Zn) levels observed across various regions. Particularly, the concentrations of Pb and Cd significantly exceed their natural background levels. P-ATPases, a group of proteins, utilize energy from ATP hydrolysis to support the transmembrane movement of metal ions. This group encompasses several Heavy Metal Associated Transporter (HMA) ATPases. Studies on hyperaccumulators have shown the critical role of HMAs in the movement and reduction in Zn and Cd toxicity in plant systems. This research identifies a protein encoded by the *SpHMA* gene from Sedum *plumbizincicola*, a species noted for aiding Zn/Cd hyperaccumulators, which enhances tolerance to Cd and Zn. We detail a protein encoded by SpH/A within the HMA family that enhances Cd tolerance. Real-time fluorescence quantification (RT-PCR) indicates that *SpHMA3* expression in *Arabidopsis thaliana* and Zea mays *KN5585* correlates with high Cd tolerance, linked to Cd accumulation in Zea mays. In addition, homozygous *Arabidopsis thaliana AtHMA3* mutants exhibited increased Cd sensitivity compared to the wild type (*WT*). Notably, plants of *Arabidopsis thaliana* and *maize* overexpressing *SpHMA3* showed enhanced Cd stress tolerance compared to *WT*. Enhanced Cd accumulation in tissues was observed when *SpHMA3* was overexpressed, as revealed by subcellular distribution analysis. We propose that *SpHMA3* augments *maize* tolerance to Cd and Zn stresses through enhanced cellular uptake and translocation of Cd ions. This investigation clarifies the gene function of *SpHMA3* in Cd and Zn stress response, offering insights for enhancing heavy metal absorption traits in *maize* varieties and phytoremediation methods for soils contaminated with heavy metals.

## 1. Introduction

Heavy metals refer to metallic elements with densities greater than 5 g/cm^3^, such as lead, mercury, cadmium, chromium, etc. They are widely found in nature, but heavy metal pollution is a growing problem due to human practices such as industrial activities, mining, and agriculture. The importance of studying heavy metals lies in their potential harm to the environment and health. Heavy metals are not easy to degrade, can accumulate in soil and water, and enter the human body through the food chain, resulting in chronic poisoning, damage to the nervous system, kidney, and immune system. In addition, heavy metal pollution can damage ecosystems and affect biodiversity. Therefore, it is very important for environmental protection and human health to study the sources, migration rules, and treatment methods of heavy metals. The swift pace of industrialization has led to significant environmental degradation, notably soil contamination with heavy metals in China, now a pressing issue [1]. Lead (Pb) and cadmium (Cd) concentrations are notably high in soils, with approximately ten percent of soil in China affected by Cd contamination, impacting farmlands across various regions to varying extents. Such contamination poses severe threats to ecological health, endangers public health, and impacts human activities and well-being [2,3]. Cd, recognized as a non-essential and harmful element, disrupts the growth of diverse biological entities [4,5]. Its inherent toxicity, persistence, and resistance to biodegradation mean even minimal exposure can culminate in significant Cd accumulation in humans, adversely affecting vital organs such as the kidneys, liver, and lungs [6]. Cd toxicity can hinder plant growth and development; high levels can cause symptoms including stunted growth, leaf and stem yellowing, reduced biomass [7], and in extreme cases, cessation of growth or plant death due to dehydration [8]. *Maize* is a crucial cereal crop globally. Since 1998, its total production has exceeded that of rice and wheat, making it the most widely produced cereal. By 2023, the global *maize* cultivation area will reach 2.04 × 10^4^ million hectares, with a production output of 1236 million tonnes [9,10,11,12,13]. Due to its high yield and adaptability compared to crops like wheat and rice, *maize* is frequently used as a model crop for research on soil heavy metal remediation. Some previous studies have indicated that lower concentrations of Cd stress did not adversely affect *maize* plants [14,15]. When exposed to higher concentrations of Cd, *maize* exhibited a marked decline in chlorophyll content, weakened photosynthetic activity, and reduced root and stem growth, ultimately leading to decreased yields [16,17].

P_IB_-type ATPases, a subfamily of P-type ATPases (HMAs), utilize the energy from ATP hydrolysis to facilitate the transmembrane transport of ions [18,19,20]. These transporters primarily mediate the transport of Zn^2+^ but are also involved in the movement of heavy metal ions harmful to plants, such as Cd^2+^ and Pb^2+^. Based on the substrate specificity, HMA transporters are classified into the Zn/Co/Cd/Pb subgroup and the Cu/Ag subgroup [21]. Studies have shown that the genomes of *Arabidopsis thaliana* and the hyperaccumulator Sedum *plumbizincicola* contain eight HMA genes, while Oryza sativa Japonica Group possesses nine HMA genes. Notably, *AtHMA1*-*AtHMA4*, *OsHMA1*-*OsHMA3*, and *SpHMA4*-*SpHMA8* belong to the Cu/Ag subgroup, whereas *AtHMA5*-*AtHMA8*, *OsHMA4*-*OsHMA9*, and *SpHMA1*-*SpHMA3* are part of the Zn/Co/Cd/Pb subgroup [21]. In *Arabidopsis thaliana*, *AtHMA1* resides within the chloroplast periplasm, facilitating the Zn expulsion from chloroplasts; the upregulation of *AtHMA3* in these plants heightens their resilience to and accumulation of Cd, Zn, Pb, and Co [22]. *AtHMA4*-deficient mutants exhibit heightened sensitivity to Cd and Zn compared to their wild-type counterparts. In the Japonica Group of Oryza sativa, *OsHMA1* plays a role in Zn transportation [23], while *OsHMA3* is exclusive to Cd transport and serves as a Cd barrier within root cell vesicles [24]. The mRNA of the *AtHMA3* protein is extensively expressed across various tissues of *Arabidopsis thaliana* and is located in the vesicular membrane, where it facilitates the transport of Cd into vesicles, thereby contributing to Cd compartmentalization. Overexpression of this gene in other plant species led to a significant increase in Cd accumulation in transgenic plants compared to wild-type plants, indicating that *AtHMA3* plays a role in Cd storage within plants [25].

Phytoremediation offers an environmentally friendly and safe approach to restoring contaminated land. Sedum *plumbizincicola*, a perennial succulent herb, thrives in areas abundant in Pb and Zn minerals [26]. However, its narrow range of adaptation, low economic value, and other limitations have significantly restricted its application and promotion. In contrast, advancements in genetic engineering to enhance plant uptake of heavy metals provide new opportunities for the phytoremediation of contaminated soils. *SpHMA3*, a heavy metal transporter found in hyperaccumulator plants, exhibits greater efficiency in transporting cadmium (Cd) and zinc (Zn) than other *HMA3* genes, and is able to more effectively sequester heavy metal ions into vacuoles, thereby enhancing plant tolerance and accumulation. The transport mechanism of *SpHMA3* may have higher substrate specificity or stronger affinity, which makes it better in the recognition and transport of heavy metal ions. In terms of practical application, *SpHMA3* shows significant potential in the field of phytoremediation. Through genetic engineering, plants can enhance the repair ability of heavy metal contaminated soil. Compared with other *HMA3* genes, *SpHMA3* has more advantages in efficiency and application prospects. This plant species is capable of extracting and hyperaccumulating Zn and Cd from soils contaminated with heavy metals, making it a viable candidate for phytoremediation of Zn and Cd contamination [27,28]. It is also important to note that traditional *maize* cultivation not only suffers yield losses due to Cd stress but also experiences a decline in kernel quality as a result of Cd presence. Therefore, enhancing Cd tolerance in *maize* and diminishing Cd accumulation in kernels significantly enhance both the yield and quality of *maize*. The HMA family, notable for its role as a heavy metal transport protein, participates in Cd transport across various plant species; however, its association with *maize* remains underexplored. This research identifies the *SpHMA3* gene as a close counterpart to the *AtHMA3* gene from *Arabidopsis*. Moreover, *Arabidopsis* mutants lacking the *AtHMA3* gene exhibited heightened Cd sensitivity. By introducing the *SpHMA3* gene into *Arabidopsis* and *maize*, plants overexpressing this transgene were developed, affirming the influence of the gene on Cd absorption and transportation. Thus, the findings from this investigation are poised to furnish a theoretical framework for employing biotechnological strategies to bolster Cd resistance in *maize* and genetically enhance the capacity of *maize* to manage Cd accumulation. At the same time, this study also provides a new bioremediation path for improving crops to solve the problem of heavy metal pollution in agricultural systems.

## 2. Results

### 2.1. Phylogenetic Tree and Conserved Structural Domain Analysis of HMA Family Genes in Several Major Crops

**Figure 1:**
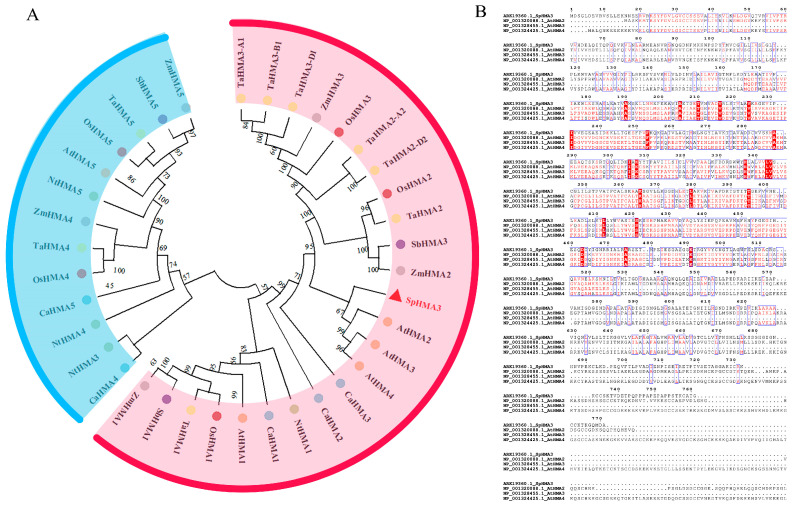
Phylogenetic construction and sequence conservation analysis of the HMA gene family (**A**) phylogenetic analysis of the HMA family in Sedum *plumbizincicola*, Zea mays, Sorghum bicolor, Oryza sativa Japonica Group, Cicer arietinum, Nicotiana *tomentosiformis*, Triticum aestivum, and *Arabidopsis thaliana*; (**B**) conservative sequence analysis of *SpHMA3* and *AtHMA2*, *3,* and *4* in *Arabidopsis thaliana*.

To obtain the CDS of the *SpHMA3* gene from Sedum *plumbizincicola* (KY312109.1), we referred to the sequence available on the NCBI database (NCBI: https://www.ncbi.nlm.nih.gov/search/all/?term=KY312109.1, accessed on 10 September 2019) and downloaded the complete sequence. To explore the evolutionary relationships between the *SpHMA3* gene of Sedum *plumbizincicola* and the HMA gene family members of Zea mays, Sorghum bicolor, Oryza sativa Japonica Group, Cicer arietinum, Nicotiana tomentosiformis, Triticum aestivum, and *Arabidopsis thaliana*, we retrieved the gene sequences of these species through NCBI (NCBI: https://www.ncbi.nlm.nih.gov/, accessed on 20 September 2019). An evolutionary tree was constructed using MEGA based on amino acid multiple sequence alignments, comparing Sedum *plumbizincicola* (*SpHMA3*) with Zea mays (*ZmHMA1*-*ZmHMA5*), Sorghum bicolor (*SbHMA1*, *SbHMA3*, *SbHMA5*), Oryza sativa Japonica Group (*OsHMA1*-*OsHMA5*), *Arabidopsis thaliana* (*AtHMA1*-*AtHMA5*), Cicer arietinum (*CaHMA1*-*CaHMA5*), Nicotiana *tomentosiformis* (*NtHMA1*-*NtHMA5*), and Triticum aestivum (*TaHMA1*-*TaHMA5*) (Figure 1A). Investigations revealed that the *SpHMA3* gene exhibits the closest relation to *AtHMA3* from *Arabidopsis thaliana*, and shares similarities with *AtHMA2* and AtHMA4 genes as well. The DNAMAN 9 software was utilized to assess sequence similarities among *SpHMA3*, *AtHMA2*, *AtHMA3* and *AtHMA4*, revealing a 52.3% homology between *SpHMA3* and *AtHMA3*. The *SpHMA3* gene was identified to possess the E1-E2 ATPase and the haloacid-like dehalogenase hydrolase structural domains, as determined by Pfam (Pfam: http://pfam.xfam.org/, accessed on 10 October 2019) and independently confirmed by SOSUI (SOSUI: https://harrier.nagahama-i-bio.ac.jp/sosui/, accessed on 23 October 2019), which also indicated eight transmembrane structural domains within *SpHMA3* (Figure 1B).

### 2.2. Identification of Arabidopsis thaliana athma3 Pure Mutants and Gene Transcription Levels

Protein sequence comparison revealed that the homologous gene to *SpHMA3* in *Arabidopsis thaliana* is *AtHMA3*. Therefore, *AtHMA3* mutant seeds were obtained from the American *Arabidopsis thaliana* Seed Resource Center (ABRC). Based on the information available from the TAIR database (https://www.arabidopsis.org/, accessed on 5 December 2022) and sequencing results, the T-DNA insertion site in the mutant was identified to be located in the promoter region of the *AtHMA3* gene, specifically within the first 155 bp upstream of the start codon ATG (Supplementary Figure S1A). The *athma3* mutant was propagated in a soil-based culture system, and its purity was verified using the three-primer method. Primers LP, RP, and LB3.1 were designed for detecting the homozygous mutant strain, and the design is available online at http://signal.salk.edu/. The purity of the *Arabidopsis thaliana athma3* mutants was confirmed to be high (Supplementary Figure S1B).

Variations in the T-DNA insertion sites may influence gene transcript levels. To explore how the homozygous loss of *SpHMA3* function impacts *Arabidopsis* under Cd stress, the *AtHMA3* gene expression in the mutant was analyzed. qT-PCR results (Supplementary Figure S1C) revealed that *AtHMA3* expression in the mutant was significantly lower, at 23.4% compared to *WT*. This suggests that the *AtHMA3* gene was only partially knocked out in the mutant.

### 2.3. Mutant of AtHMA3 Decreased Resistance to Cd Stress in Arabidopsis

**Figure 2:**
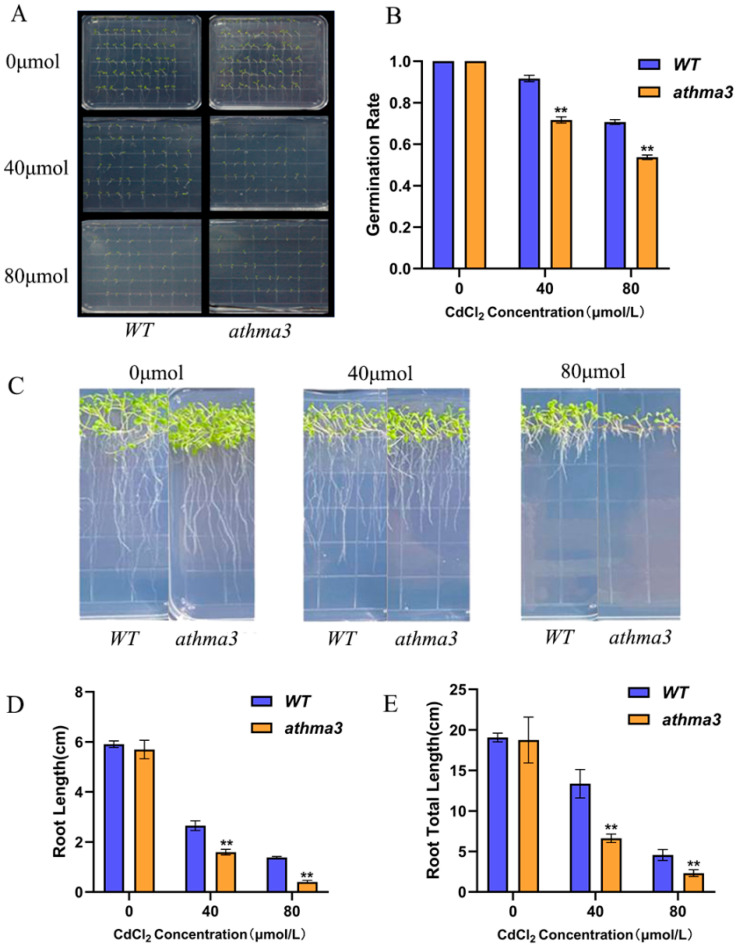
Analysis of phenotypic and root characteristics in *Arabidopsis thaliana AtHMA3* homozygous mutants: (**A**) Comparison of phenotype between *Arabidopsis thaliana AtHMA3* homozygous mutants and *WT* during germination exposed to varying Cd concentrations; (**B**) Germination rates for both *Arabidopsis thaliana AtHMA3* homozygous mutants and *WT* seeds assessed on day 7 across different Cd stress levels; (**C**) Graphical representation depicting variations in root length and overall root system development in *Arabidopsis thaliana AtHMA3* homozygous mutants and *WT* under varied Cd concentrations; (**D**,**E**) Charts displaying primary root length and total root system extent under Cd stress conditions; ** denotes *p* < 0.01; analyzed using Student’s *t*-test.

To investigate the impact of varying Cd stress concentrations on the growth of *Arabidopsis thaliana* mutant seedlings, three CdCl_2_ concentrations were used: 0 μmol/L, 40 μmol/L, and 80 μmol/L, with parallel experiments conducted for each. Germination rates were recorded on the 7th day (Figure 2B). Under normal conditions, the germination rate of *athma3* mutants was comparable to *WT* at 100%. Under 40 μmol/L CdCl_2_ stress, the germination rate of the mutant decreased to 71.7%, whereas that of WT was 91.7%. When the stress concentration was increased to 80 μmol/L, the germination rate of the mutant further decreased to 53.9%, while that of *WT* was 70.6% (Figure 2A). Under Cd stress, root length of *athma3* mutants was significantly shorter than *WT*. Root lengths were measured and showed notable differences between the mutant and *WT* (Figure 2C). Under non-stress conditions, primary root length of the *athma3* mutant was similar to *WT*. Under 40 μmol/L CdCl_2_ stress, the primary and total root lengths of *WT* were 1.83 and 1.94 times that of the *athma3* mutant. At 80 μmol/L CdCl_2_, these values increased to 3.97 and 3.28 times, respectively (Figure 2D,E). The inhibition of root length became more pronounced with higher Cd concentrations, indicating that the *AtHMA3* gene plays a crucial role in the response of *Arabidopsis thaliana* to Cd stress.

### 2.4. Decreased Cd Accumulation in the Arabidopsis thaliana athma3 Mutant

**Figure 3:**
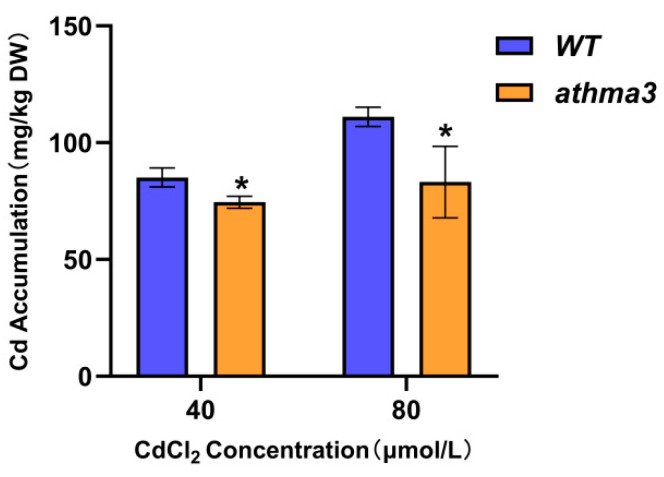
Variation in Cd levels in *Arabidopsis thaliana athma3* mutant and *WT* under varying Cd stress conditions; * denotes *p* ≤ 0.05; analyzed using Student’s *t*-test.

To examine the capability of *Arabidopsis thaliana athma3* mutant plants to accumulate Cd under Cd stress conditions, experiments were performed using Cd stress at concentrations of 40 μmol/L and 80 μmol/L. Both *WT* and *athma3* mutant seeds were sterilized and cultivated on 1/2MS medium supplemented with varying gradients of CdCl_2_ for 14 days. *Arabidopsis thaliana* seedlings subjected to different Cd treatments were collected, dried, and weighed for nitrification, and their Cd content was measured using an inductively coupled plasma mass spectrometer. The results showed that the *athma3* mutant had lower Cd content compared to *WT* under both stress concentrations. Under the 40 μmol/L treatment, the Cd content in the *athma3* mutant was 87.6% of that in the *WT*, while under the 80 μmol/L treatment, it was 74.8% of that in the *WT* (Figure 3). These findings suggest that a significant reduction or loss of *AtHMA3* gene expression resulted in decreased Cd accumulation in the plants.

### 2.5. Overexpression of ZmHMA3 Increases Tolerance to Heavy Metal Stress in Arabidopsis thaliana

**Figure 4:**
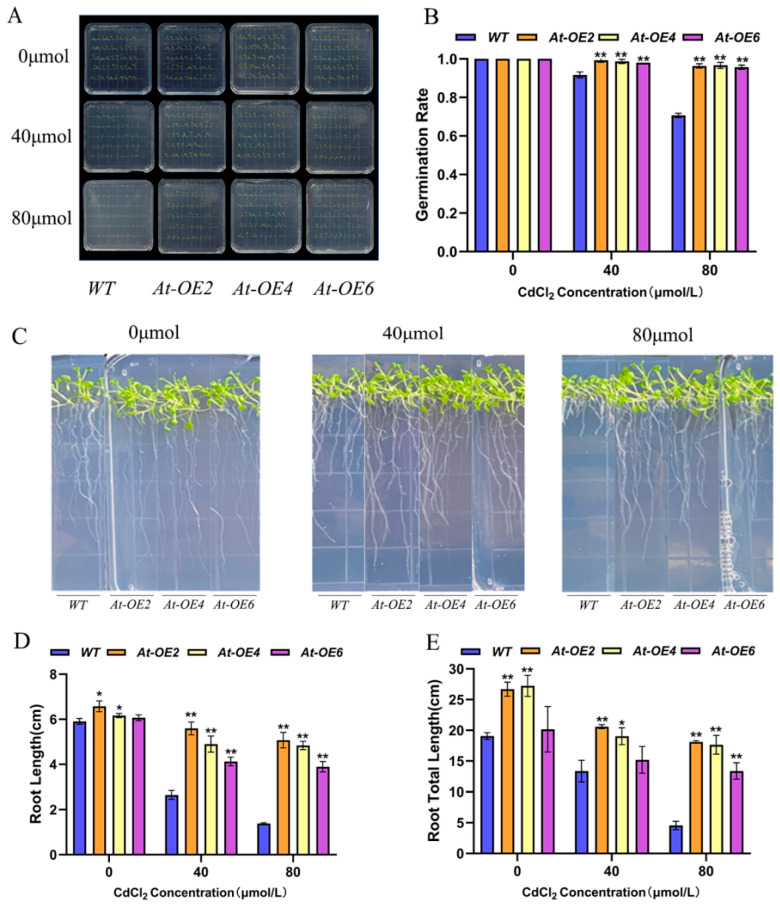
Phenotypic characterization and root trait analysis of *Arabidopsis thaliana* overexpressing *SpHMA3* gene transformation events: (**A**) Seventh-day germination phenotypes of *Arabidopsis thaliana SpHMA3* overexpression materials and *WT* seeds under varying Cd stress concentrations; (**B**) Seventh-day germination rates of *Arabidopsis thaliana SpHMA3* overexpression materials and *WT* seeds under varying Cd stress concentrations. (**C**) Identification of root growth of *Arabidopsis thaliana SpHMA3* overexpression and *WT* plants at 14 d under varying Cd stress concentrations; (**D**) Determination of primary root length of *Arabidopsis thaliana SpHMA3* overexpression material and *WT* seeds at 14 d under varying Cd stress concentrations; (**E**) Determination of total root length of *Arabidopsis thaliana SpHMA3* overexpression material and *WT* seeds at 14 d under varying Cd stress concentrations; * denotes *p* ≤ 0.05; ** denotes *p* ≤ 0.01; analyzed using Student’s *t*-test.

To investigate the role of *SpHMA3* in response to Cd stress, the *SpHMA3* gene was introduced into *Arabidopsis thaliana*, resulting in nine transgenic lines, designated *SpHMA3OE1*-*SpHMA3OE9*, later renamed *AtOE1*-*AtOE9*. During subsequent cultivation, due to sterility issues, only three lines, *AtOE2*, *AtOE4*, and *AtOE6*, were retained for further study. The germination rates and agronomic characteristics of these transgenic *Arabidopsis* seedlings were assessed under various heavy metal stress conditions (Figure 4A–E).

Seeds of both *Arabidopsis thaliana* overexpression lines (*OE*) and *WT* were germinated on 1/2MS medium containing CdCl_2_ concentrations of 0 μmol/L, 40 μmol/L, and 80 μmol/L in three sets of parallel experiments, with germination rates recorded on day 7. Under no-stress conditions, both the *WT* and *OE* plants grew normally. Cd stress reduced germination in both plant types, though the *OE* lines exhibited higher germination rates compared to *WT* under stress (Figure 4A). Under 40 μmol CdCl_2_ stress, the germination rates of *At-OE2*, *At-OE4*, and *At-OE6* were 99.4%, 98.9%, and 98.3%, respectively, while the *WT* showed a rate of 91.7%. Under 80 μmol CdCl_2_ stress, *At-OE2*, *At-OE4*, and *At-OE6* had germination rates of 96.1%, 96.7%, and 95.6%, respectively, with the *WT* at 70.6% (Figure 4B). Meanwhile, after 14 days of growth under the same conditions, the root lengths of transgenic and *WT* plants exposed to varying Cd concentrations were measured. Overexpression of the *SpHMA3* gene enhanced seedling growth, particularly under Cd stress, showing more pronounced growth compared to the *WT* (Figure 4C). Under 40 μmol CdCl_2_ stress, the primary roots of *At-OE2*, *At-OE4*, and *At-OE6* were 2.11, 1.85, and 1.56 times longer than those of the *WT*, while total root lengths were 1.54, 1.43, and 1.14 times longer. At 80 μmol CdCl_2_ stress, the primary roots of *At-OE2*, *At-OE4*, and *At-OE6* were 3.67, 3.5, and 2.72 times longer, with total root lengths 3.98, 3.87, and 2.93 times longer than the *WT* (Figure 4D,E). This indicates that when the *Arabidopsis thaliana SpHMA3* gene was overexpressed, the overexpression plants showed better tolerance and environmental adaptation under Cd stress compared with the *WT*.

### 2.6. Overexpression of SpHMA3 Improves Tolerance to Heavy Metal Stress in Maize

**Figure 5:**
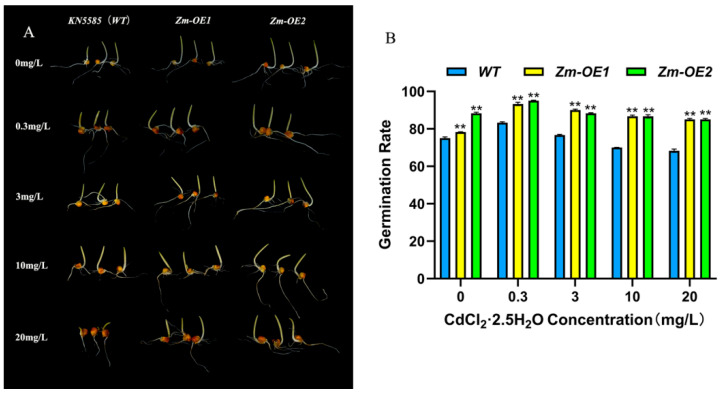
Germination of *WT* and *OE* seeds under Cd stress conditions: (**A**) Seed germination comparison between *WT* and *OE* under varying Cd stress concentrations; (**B**) Statistical analysis of seed germination rates for *WT* and *OE* under varying Cd stress concentrations; ** denotes *p* ≤ 0.01; analyzed using Student’s *t*-test.

To determine the function of *SpHMA3* in *maize* in response to Cd stress, we transformed the *SpHMA3* gene into Zea mays *KN5585* (*WT*) and obtained a total of two transgenic *maize* materials, *SpHMA3-OE1* and *SpHMA3-OE2*, named *Zm-OE1* and *Zm-OE2*. We compared the differences in germination rates between overexpressed *maize* and the *WT* under different heavy metal stresses (Figure 5A,B). The *KN5585* (*WT*) and *OE* plants were exposed to varying concentrations of CdCl_2_·2.5H_2_O solutions, specifically 0.3 mg/L, 3 mg/L, 10 mg/L, and 20 mg/L, for a set period. A comparison of growth phenotypes between *KN5585* and *OE* plants is illustrated in Figure 5B. It is observed that the seed germination rates of *WT*, *Zm-OE1*, and *Zm-OE2* initially increased and then decreased as the Cd stress concentration rose. The highest germination rates, 83.33%, 93.33%, and 95.00%, respectively, were recorded at 0.3 mg/L Cd, while the lowest rate, 68.33%, was seen at 20 mg/L. As the Cd concentration increased, *OE* plants consistently showed significantly higher germination rates than the *WT*, indicating better adaptation to Cd stress. These results suggest that Cd stress considerably inhibits *WT* seed germination, while the *OE* material may possess a protective mechanism that reduces Cd toxicity, thereby enhancing plant survival.

### 2.7. Functional Expression of SpHMA3 Under Cd Stress

**Figure 6:**
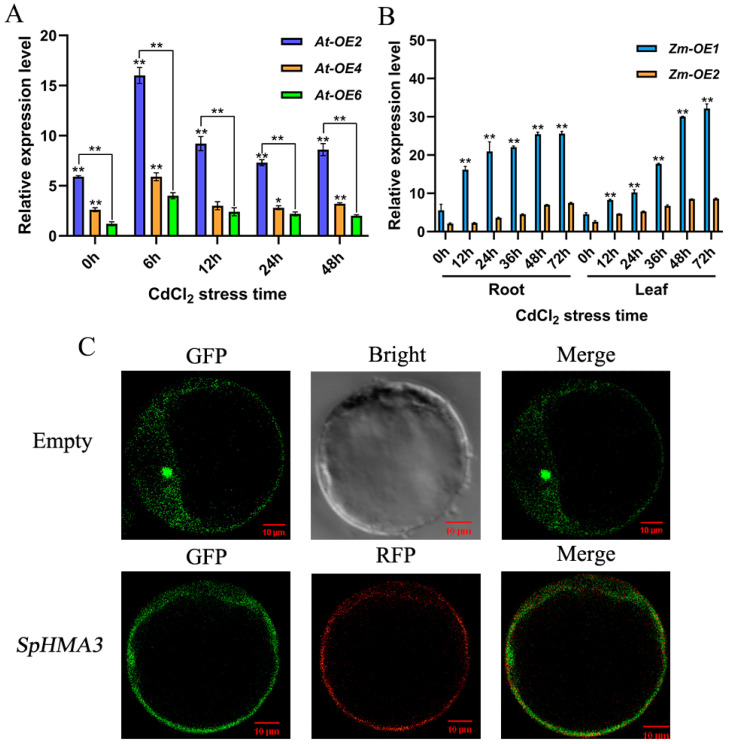
Tissue-specific expression and subcellular localization of *SpHMA3* in *maize* and *Arabidopsis thaliana*: (**A**) Analysis of *SpHMA3* expression in converted material of *Arabidopsis thaliana*; (**B**) Analysis of *SpHMA3* expression in various tissues of *maize*; (**C**) Observation of the localization of *SpHMA3* in *maize* protoplasts; * denotes *p* ≤ 0.05; ** denotes *p* ≤ 0.01; analyzed using Student’s *t*-test. Note: (**A**), Graphs present the relative expression levels of the *SpHMA3* gene in the entire *Arabidopsis thaliana* plant at 6, 12, 24, and 48 h after exposure to 80 μmol/L Cd stress; (**B**), Graphs depict the relative expression of the *SpHMA3* gene in *maize* root and leaf tissues subjected to 12, 24, 36, 48, and 72 h of Cd stress at a concentration of 800 μmol/L; (**C**), GFP server represents green fluorescent protein, the RFP server refers to red fluorescent protein, the bright field image shows cell morphology, and the merged image integrates dark fluorescence with cell structure. The scale bar represents 10 μm.

To investigate the function of *SpHMA3* under Cd stress, we examined the relative expression levels in transgenic *maize* subjected to Cd stress. When exposed to 80 μmol/L CdCl_2_, RNA samples from *Arabidopsis thaliana* transformation events *At-OE2*, *At-OE4*, and *At-OE6* were collected at various time points for analysis of *SpHMA3* expression using qT-PCR (Figure 6A). Under 800 μmol/L Cd stress, RNA was extracted from the roots and leaves of transgenic Zea mays lines *Zm-OE1* and *Zm-OE2*, and the *SpHMA3* expression patterns were determined via qRT-PCR, with untreated plants serving as the control (Figure 6B). Subcellular localization predictions for *SpHMA3*, performed using Softberry (http://www.softberry.com/, accessed on 10 September 2020), indicated a strong likelihood of localization in the cytoplasmic membrane based on homology to known localization genes in LocDB, suggesting that *SpHMA3* might reside in the cell membrane. To verify this conclusion, the recombinant plasmid was introduced into *maize* protoplasts alongside the cytoplasmic marker vector PM-RFP. After 12 h of expression, fluorescence confocal microscopy revealed green fluorescence on the cytoplasmic membrane, with yellow fluorescence observed after co-localization with the marker vector (Figure 6C), confirming that *SpHMA3* is localized to the *maize* cell membrane.

### 2.8. Overexpression of the SpHMA3 Gene Enhances the Accumulation Enrichment Capacity of Plants to Heavy Metal Stresses

**Figure 7:**
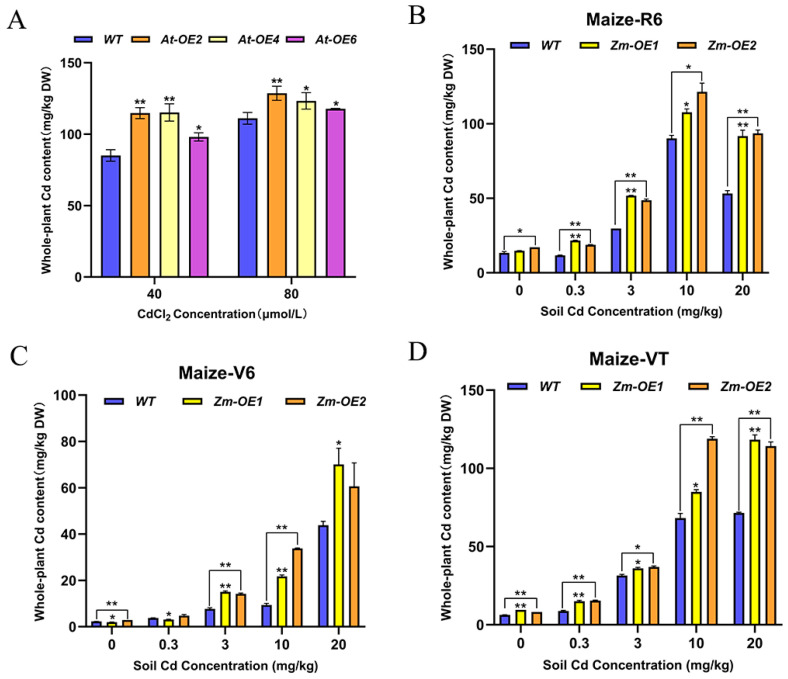
Comparison of the differences in the ability of *WT* and *SpHMA3* overexpression on Cd accumulation: (**A**) Cd content of *Arabidopsis thaliana SpHMA3* overexpression plants and *WT*; (**B**) Cd content of *maize SpHMA3* overexpression plants and *WT* in whole plant of *Maize*-R6; (**C**) Cd content of *maize SpHMA3* overexpression plants and *WT* in whole plant of Maize-V6; (**D**) *maize SpHMA3* overexpressing plants and *WT* in Maize-VT whole plant Cd content; * denotes *p* ≤ 0.05; ** denotes *p* ≤ 0.01; analyzed using Student’s *t*-test.

**Figure 8:**
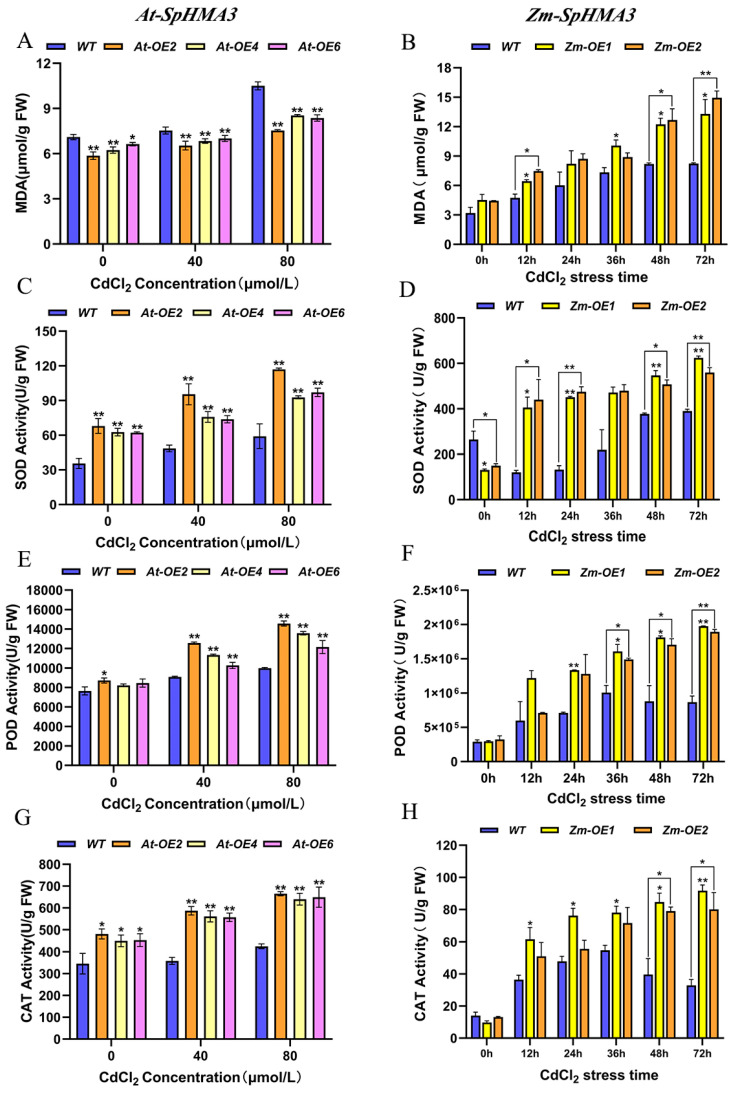
Physiological and biochemical expression level analysis of *SpHMA3* transgenic *maize* and *Arabidopsis thaliana* under Cd stress conditions: (**A**) Influence of Cd stress on MDA levels in *Arabidopsis thaliana* MDA; (**B**) Influence of Cd stress on MDA levels in *maize*; (**C**) Influence of Cd stress on SOD activity in *Arabidopsis thaliana*; (**D**) Influence of Cd stress on SOD activity in *maize*; (**E**) Influence of Cd stress on POD activity in *Arabidopsis thaliana*; (**F**) Influence of Cd stress on POD activity in *maize*; (**G**) Influence of Cd stress on CAT activity in *Arabidopsis thaliana*; (**H**) Influence of Cd stress on CAT activity in *maize*; * denotes *p* ≤ 0.05; ** denotes *p* ≤ 0.01; analyzed using Student’s *t*-test.

The *SpHMA3* gene plays a crucial role in Cd accumulation in the hyperaccumulator Sedum *plumbizincicola*. To evaluate the Cd enrichment potential of transgenic materials developed by introducing this gene into *maize* and *Arabidopsis thaliana*, Cd content was measured and compared between the transgenic lines and wild types under Cd stress conditions. *WT*, *At-OE2*, *At-OE4*, and *At-OE6* were grown in media containing 40 μmol/L and 80 μmol/L CdCl_2_, and whole-plant Cd content was assessed after 14 days (Figure 7A). Meanwhile, *maize WT* and *OE* plants were cultivated in soil containing Cd at concentrations of 0 mg/kg, 0.3 mg/kg, 3 mg/kg, 10 mg/kg, and 20 mg/kg. Samples were taken for Cd content analysis during the nodulation stage (Maize-V6), stamen withdrawal (Maize-VT), and maturity (Maize-R6) (Figure 7B–D). The data demonstrated that Cd content in *Arabidopsis thaliana* overexpression plants was consistently higher than in *WT* under both concentration treatments (Figure 8A). The Cd levels in *At-OE2*, *At-OE4*, and *At-OE6* at 40 μmol/L were 1.35, 1.34, and 1.15 times higher, respectively, than in *WT*, while at 80 μmol/L, the increases were 1.16, 1.11, and 1.06 times, respectively. In *maize*, Cd levels in overexpression plants surpassed those in *WT* at all tested concentrations (Figure 7B–D). During the Maize-V6 period, Cd levels in *OE* plants remained low, but a significant increase was observed in the Maize-VT period, suggesting enhanced Cd uptake and translocation in *OE* plants. Across all Cd treatments, *OE* plants consistently showed higher Cd accumulation than the *WT*, indicating that the *SpHMA3* gene markedly enhances Cd enrichment in *maize*.

### 2.9. Physiological Response of SpHMA3 Transgenic Maize and Arabidopsis Under Cd Stress

To examine the physiological and biochemical responses of transgenic *Arabidopsis thaliana* plants under Cd stress, both *WT* and *OE Arabidopsis thaliana* were cultivated in media containing 40 μmol/L and 80 μmol/L CdCl_2_ for 14 days. The MDA content and activities of the enzymes CAT, POD, and SOD were measured (Figure 8A,C,E,G). Meanwhile, transgenic *maize* and *WT* plants were evaluated, with *maize WT* and *OE* grown to the three-leaf stage and then subjected to 80 μmol/LCdCl_2_ solution for 0, 12, 24, 36, 48, and 72 h. Enzymatic activities and MDA content were assessed in the root systems of *maize* (Figure 8B,D,F,H). As seen in Figure 8A,B, under Cd stress, the MDA level in *OE Arabidopsis thaliana* was lower than in *WT*, while the transgenic *maize* showed higher MDA levels than *WT* at certain times. This variation might be due to differences in stress concentration and gene expression. Both *Arabidopsis thaliana* and *maize* demonstrated an upward trend in MDA content as Cd concentration and stress duration increased, consistent with the known effect of heavy metal stress on MDA accumulation. Figure 8C–H illustrate that CAT, POD, and SOD enzyme activities increased under Cd stress in both transgenic and *WT* plants. In particular, the enzymatic activities in transgenic *Arabidopsis thaliana* were more pronounced under 80 μmol/L CdCl_2_ than under 40 μmol/L CdCl_2_ (Figure 8C,E,G). A similar pattern was observed in *maize*, where CAT, POD, and SOD enzyme activities increased over time in transgenic *maize*, reaching significantly higher levels compared to *WT* under certain stress conditions (Figure 8D,F,H). These findings suggest that the expression of the *SpHMA3* gene was enhanced as stress levels intensified, leading to increased antioxidant enzyme activity, which likely played a role in protecting the cells from stress.

### 2.10. Remediation of Cd-Contaminated Soil by Overexpressing SpHMA3 Maize

**Figure 9:**
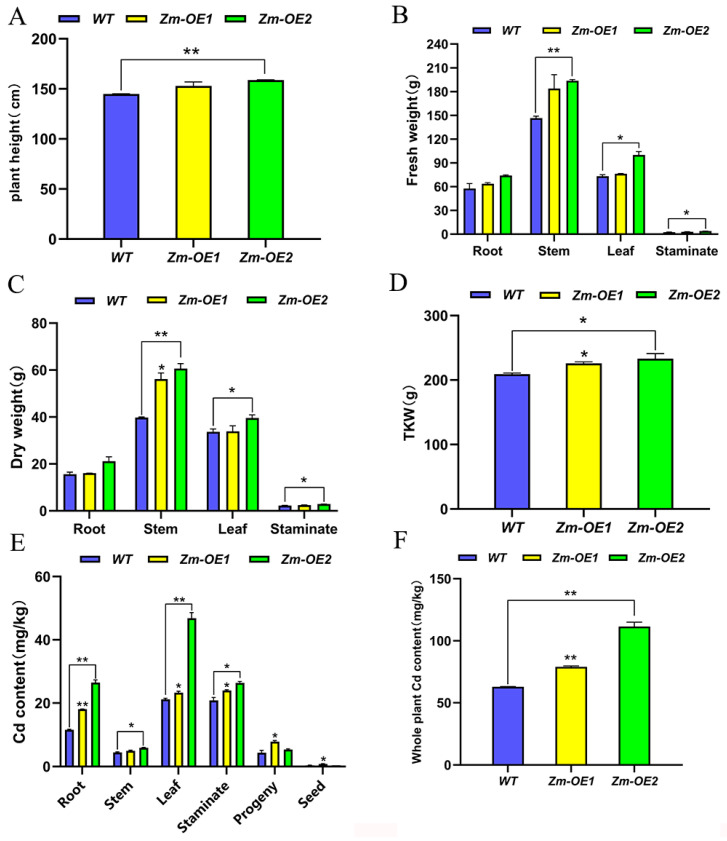
Changes in agronomic traits and Cd content of *maize* on Cd-contaminated soil: (**A**) Changes in *maize* plant height; (**B**) Fresh weight distribution across different *maize* parts; (**C**) Dry weight accumulation in various parts of *maize*; (**D**) Thousand-kernel weight comparison; (**E**) Examination of Cd distribution across *maize* parts; and (**F**) Comparison of the accumulation of Cd by the material as a whole; * denotes *p* ≤ 0.05; ** denotes *p* ≤ 0.01; analyzed using Student’s *t*-test.

*Maize*, as the most widely cultivated crop, exhibits significantly enhanced adaptability to Cd environments when *SpHMA3* is overexpressed. Prior research has indicated that *maize* can absorb Cd from contaminated environments, contributing to the reduction in soil Cd levels. To explore whether *SpHMA3*-overexpressing *maize* could be utilized for soil Cd remediation, a study was conducted. In Cd-contaminated farmland (pH 6.87, Cd concentration 14.6 mg/kg), *WT* and *OE KN5585 maize* plants were grown under standard field conditions. During the maturation stage, data on plant height, and fresh/dry weights of roots, stems, leaves, male ears, and thousand kernel weights (TKW) were collected (Figure 9A–D). Results showed that *Zm-OE1* and *Zm-OE2* plants exhibited greater plant height and higher fresh/dry weights of leaves, stems, and male ears compared to *WT* under Cd stress (Figure 9A–C). Although Cd treatment reduced TKW for both *WT* and *OE* materials, the *OE* consistently maintained higher TKW than the *WT*, indicating superior bioaccumulation (Figure 9D). *Zm-OE2* outperformed the *WT* in all measured traits, while *Zm-OE1* surpassed the *WT* in all traits except female spikes under Cd stress. Further analysis of Cd content revealed that *Zm-OE1* and *Zm-OE2* accumulated more Cd in leaves, roots, stems, male spikes, female spikes, and seeds than *WT*. Among them, in the root system, the Cd contents of *SpHMA3-OE1* and *SpHMA3-OE2* were highly significant higher than that of *WT*, elevated by 55.12% and 127.37%, respectively. The Cd content in the stalk, leaves, female and male ears also showed significantly higher overexpression material than the *WT* (Figure 9E). Determining the Cd content of the whole *maize* plant, we found that the Cd content of *Zm-OE1* and *Zm-OE2* was extremely significantly higher than that of *WT* by 89.14% and 166.87%, respectively (Figure 9F), suggesting that *SpHMA3* overexpression materials are more suitable for growth in the Cd environment than the *WT*.

### 2.11. Characterization of Cd Enrichment Transfer in SpHMA3 Overexpression Material

**Figure 10:**
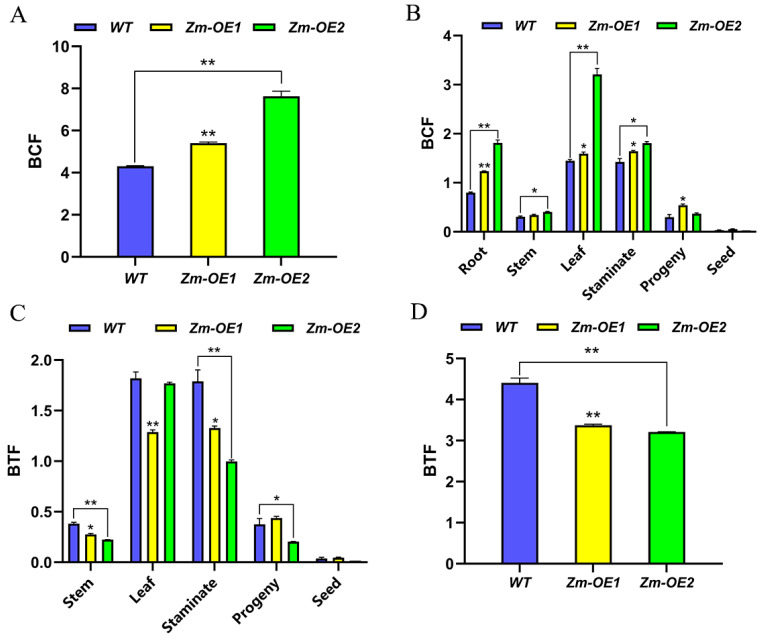
Enrichment and translocation capacity of *maize* for soil Cd determined by: (**A**) enrichment capacity of different parts of *maize* for Cd; (**B**) enrichment capacity of *maize* monocultures for Cd; (**C**) translocation capacity of different parts of *maize* for Cd; and (**D**) translocation capacity of *maize* monocultures for Cd; * denotes *p* ≤ 0.05; ** denotes *p* ≤ 0.01; analyzed using Student’s *t*-test.

Based on the above studies, we analyzed the enrichment coefficients (BCF) and transfer coefficients (BTF) of *WT* and overexpressed *maize* materials for soil Cd in various tissues, organs, and the whole of the *maize* plant under Cd farmland growing conditions. Figure 10A shows that the overexpression of the *SpHMA3* gene in *maize* led to higher BCF in various tissues compared to *WT*, particularly in the root system, where *Zm-OE1* and *Zm-OE2* exhibited Cd BCF that were significantly higher than *WT* by 55.12% and 127.37%, respectively. This demonstrates that *maize* roots have a stronger capacity for Cd enrichment compared to other tissues, and the absorbed Cd was primarily concentrated in the roots. *SpHMA3*-overexpressing *maize* monocotyledons showed a significantly greater Cd enrichment ability than the *WT*, with an increase of 1.26 and 1.77 times, respectively (Figure 10B). The analysis of Cd translocation in *maize* monocots revealed that the *WT* exhibited a stronger ability to transfer Cd than *OE* lines (Figure 10C). According to Figure 10D, most of the Cd absorbed by the roots was transported to the leaves, and it was notable that *WT* had a higher capacity to move Cd from the roots to the leaves, stems, and male ears compared to the *OE* lines. This may result in increased Cd levels in the aerial parts of the plant, potentially impacting photosynthesis. For a hyperaccumulator, both the BCF and BTF should exceed 1.0. In this study, both BCF and BTF in *SpHMA3*-overexpressing *maize* exceeded 1.0, indicating that while this transgenic *maize* had a higher Cd enrichment capacity than the *WT*, its translocation ability was lower. This suggests that the overexpressed *SpHMA3* gene enhanced the absorption of Cd from the soil and its retention in the root system, thereby limiting its movement to the aerial parts and reducing toxicity in those tissues. Thus, this transgenic material holds potential as a hyperaccumulator for remediating Cd-contaminated soils.

## 3. Discussion

*SpHMA3* was obtained from Zn- and Cd-hyperaccumulating plants located in southeastern China. It is widely acknowledged that most *HMA* proteins are involved in metal transportation, including Cd transport in vivo. Previous research indicated that *SpHMA3* functions as a P-type ATPase heavy metal transporter, enabling Sedum species to accumulate high levels of Cd in their shoots without exhibiting toxicity [29,30]. Cadmium is a highly toxic non-essential element for all organic life forms, including plants and humans. Under Cd stress, plants have evolved multiple protective mechanisms, such as Cd^2+^ chelation, vesicle sequestration, regulation of Cd^2+^ uptake, and enhanced antioxidant defense [31]. By isolating the *SpHMA3* homolog from the Cd hyperaccumulating *S. plumbizincicola*, a genetic transformation system was established between this plant and the non-hyperaccumulating ecotype *S. alfedii*, allowing for the quantification of its expression in various tissues under Cd stress [32]. Although the role of *SpHMA3* has been confirmed in organisms such as yeast, southeastern Sedum, and Chinese cabbage, its function in *maize* under Cd stress remains unexplored. Structural analysis revealed that *SpHMA3* shares significant homology with other *HMA3* proteins and shows greater similarity to *AtHMA2*, *AtHMA3*, and *AtHMA4* in *Arabidopsis thaliana* (Figure 1A). *SpHMA3* is part of the Zn/Cd subclass and contains eight transmembrane regions, including two conserved domains, the E1-E2 ATPase and, haloacid dehalogenase hydrolase domains (Figure 1B).

Subcellular localization analysis of *maize* protoplasts revealed that the *SpHMA3* gene was positioned on the cytoplasmic membrane (Figure 6C), which contrasts with its localization on the vesicular membrane in Sedum species [32]. This difference is likely attributed to species-specific variations, resulting in the same gene performing its function in distinct subcellular structures across different species. The results of this study are similar to those of a previous study on the localization of *ZmHMA3* genes in *maize* [33], which are located on cell membranes and are associated with functions such as the transfer and transport of heavy metals in and out of the cell.

Research on the *AtHMA3* gene has demonstrated that overexpression of this gene enhances plant tolerance to Cd, whereas the deletion of *AtHMA3* reduces tolerance to Cd [34]. *AtHMA3* is primarily expressed in the vacuole membrane, and its mechanism of action involves sequestering heavy metals into vacuoles, thereby reducing toxicity to other organelles. The seed germination stage is particularly sensitive to external environmental factors, and heavy metal stress during this phase can negatively impact plant growth and biomass accumulation in later stages. Under Cd stress, the *AtHMA3*-deficient mutant exhibited a notably lower germination rate compared to the *WT* (Figure 2A). Cd toxicity primarily affects plant roots by damaging root cell membranes, which impairs normal root development, as evidenced by inhibited root elongation. In this study, the root length of the *AtHMA3*-deletion mutant was significantly more inhibited under Cd stress (Figure 2C), consistent with previous findings [24] that show increased sensitivity to Cd in the *athma3* mutant. By measuring the Cd content in the mutant and *WT*, we found that the Cd content in the *athma3* mutant is significantly lower than that in *WT*, which indicates that the *HMA* gene affects the uptake and accumulation of Cd in the plant.

In recent years, substantial research has been conducted on the physiological functions of *HMA3* in non-accumulating species, such as *AtHMA3* in *Arabidopsis thaliana* and *OsHMA3* in Oryza sativa Japonica Group. Overexpression of *AtHMA3* or *OsHMA3* in these species significantly enhanced tolerance to toxic Cd [24,34]. In this study, plants expressing *SpHMA3* in *Arabidopsis thaliana* and *maize* exhibited higher Cd accumulation under Cd treatment (Figure 7), and the *SpHMA3* gene showed increased expression in roots and leaves during Cd stress (Figure 6A). Although overall seed germination rates decreased with rising Cd concentrations, *SpHMA3*-expressing plants demonstrated a significantly higher germination rate, indicating improved Cd stress resistance in these plants. Root growth analysis further revealed that under Cd stress, *SpHMA3*-expressing plants experienced less inhibition compared to the *WT*, with better root development and more fully extended lateral roots. These results suggest that *SpHMA3* expression helps mitigate the adverse effects of Cd on root systems, thereby enhancing Cd tolerance [35].

Plant cells possess a variety of antioxidant enzyme systems. Under stress conditions, the levels of reactive oxygen species in the cell increase, leading to higher H_2_O_2_ concentrations and cellular imbalance. Antioxidant enzymes, such as SOD, POD, CAT, along with some small molecule metabolites, help eliminate free radicals and reduce H_2_O_2_ levels [36]. Once Cd enters the cell, it binds to Cys residues, which can deactivate enzymes [34] and disrupt redox balance, causing oxidative stress. The content of MDA, a biomarker for oxidative stress, reflects the degree of lipid peroxidation in the cell and indicates the ability of the plant to adapt under stress conditions. In this study, examining the activities of antioxidant enzymes in *SpHMA3*-overexpressing *Arabidopsis* and *maize* under Cd stress revealed that MDA content increased in both *WT* and *OE* plants. However, *WT* plants showed a larger increase in *Arabidopsis*, whereas the opposite was observed in *maize* (Figure 8A,B). This disparity could be attributed to higher Cd accumulation in *OE maize* plants compared to the *WT*, which results in greater MDA levels in *OE* plants. This higher Cd accumulation in *OE* plants could further stimulate the activities of related enzymes. The activities of SOD, POD, and CAT were also measured in both *Arabidopsis* and *maize*, showing that *SpHMA3* overexpression significantly enhanced antioxidant enzyme activities compared to *WT* plants (Figure 8C–H). This suggests that *SpHMA3* expression strengthens antioxidant defenses, thereby mitigating Cd-induced free radical damage. Considering the higher Cd accumulation in *OE* plants compared to the *WT*, as seen in Figure 7, it can be concluded that overexpression of SpHMA3 enhances Cd uptake, translocation, and accumulation in plants, which in turn promotes gene expression and increases antioxidant enzyme activity, thereby improving the tolerance of plants to Cd. These results are consistent with previous findings [37,38]. Studies have demonstrated that under Cd^2+^ stress, NH_4_^+^ alleviates cadmium toxicity in rice seedlings by activating the *bZIP20-APX2/CATA* transcription module in an ABA-dependent manner, indicating that different species have evolved a rich array of mechanisms to resist environmental stress during their evolutionary process [39]. With the global spread of *maize*, significant potential exists for its further development. This study demonstrates that transgenic *maize*, modified with the *SpHMA3* gen, exhibits superior Cd tolerance and transfer properties compared to conventional varieties (Figure 9 and Figure 10). In recent years, numerous successful cases of soil bioremediation have been reported, and the transgenic *maize* in this research could serve as a reference for future remediation of Cd-contaminated soils [40,41]. Meanwhile, the remediated *maize* can be utilized as a biofeedstock for ethanol fermentation, promoting a sustainable, low-carbon, and environmentally friendly development model [42]. These results deepen our understanding of the function of *SpHMA3* in enhancing *maize* growth and resilience to Cd stress. Of course, it is worth noting that the premise of using corn as a soil cadmium pollution remediation plant for bioremediation is to enhance people’s recognition of this remediation model. The key lies in how to make corn complete soil restoration and realize sustainable utilization of corn. This is also the starting point of the current research work. We firmly believe that in the future, there will be many successful cases like genetically modified corn to repair cadmium pollution, and this model will also provide a new green way for soil remediation. This research provides valuable insight into improving the metal accumulation traits of current *maize* varieties and advancing phytoremediation technology for heavy metal-polluted soils.

## 4. Materials and Methods

### 4.1. Plant Materials and Growing Conditions

The *maize* inbred line *KN5585* was supplied by Sichuan Agricultural University, while the Colombian wild-type *Arabidopsis thaliana* and *AtHMA* mutant were sourced from ABRC. Transient expression receptor *maize* and *maize* healing tissues were provided by the group of Haijian Lin at the Maize Research Institute, Sichuan Agricultural University. The E. coli receptor Trans1-T1, Agrobacterium receptor GV3101, *Arabidopsis* expression vector pRI101-AN, tobacco vector pCAMBIA2300-35S-eGFP for transient expression, and *maize* expression vector pCAMBIA3300 were also obtained from the group of Haijian Lin. *Arabidopsis* plants were grown in a short-day incubator for two months before the imbibed seeds were collected and cultured on 1/2 MS solid medium containing CdCl_2_. Smut was grown in pots and exposed to light for 3 to 4 weeks and used for subcellular localization studies. The soil utilized in this study was sourced from an agricultural site in Chengdu, Sichuan Province, China. The physical and chemical properties of the soil were characterized as follows: the pH value was 7.32, the organic matter content was 46.10 g/kg, the alkali-hydrolyzed nitrogen content was 80.0 mg/kg, the available phosphorus content was 62.8 mg/kg, and the available potassium content was 182.6 mg/kg.

### 4.2. SpHMA3 and AtHMA3 Expression Analysis

Expression profiles were analyzed using an ABI 7500 real-time PCR system (Torrance, CA, USA). *KN5585 maize* seedlings were exposed to 800 μmol/L CdCl_2_, while *Arabidopsis* seedlings were treated with 80 μmol/L CdCl_2_. The CdC1_2_·2.5H_2_O used in the study was provided by Shanghai Test Reagent. Total RNA from *KN5585 maize* seedling roots, leaves, and *Arabidopsis thaliana* was isolated using TRIzol reagent (Invitrogen, Gaithersburg, MD, USA). RNA integrity was assessed through 1% agarose gel electrophoresis, and RNA concentration was measured by a nucleic acid protein assay. The cDNA sequence of the *SpHMA3* gene was retrieved from NCBI, and after designing primers via Gramene, the sequence was amplified using Novizime high-fidelity enzyme. The primers used are listed in Table S1, while the PCR mix and reaction conditions are shown in Tables S2 and S3. The ABclonal Biologicals ABScript III RT Master Mix for qPCR, coupled with the gDNA Remover kit, and the Nearshore Protein NovoScript^®^Plus All-in-one 1st Strand cDNA Synthesis SuperMix (gDNA Purge) kit were utilized to reverse transcribed RNA into cDNA at a concentration of 1000 ng/μL^−1^. The CDS of the *SpHMA3* gene (gene ID: KY312109) was obtained from NCBI and used for further analysis. The sequence was analyzed for homologous comparison, quantitative primer design, and primer specificity verification using the website (https://Plants.ensembl.org/Zea_mays/Info/Index?db=core, accessed on 20 March 2024). Primer specificity was assessed via 1% agarose gel electrophoresis, using GAPDH as the internal reference. The primer sequences are listed in Table S4, the quantitative PCR setup is provided in Table S5, and the reaction protocols are detailed in Table S6. Each experiment included three replicates, and relative gene expression levels of *SpHMA3* and *AtHMA3* were determined by the 2^−ΔΔCT^ method.

### 4.3. Subcellular Localization Assay

The subcellular localization of target proteins was predicted using the online software Softberry (https://www.softberry.com/, accessed on 22 February 2023). *SpHMA3* from Companion Mine Sedum was cloned into pCAMBIA2300-35S-eGFP, and the positive expression vector was transformed into E. coli Trans1-T1 receptor cells. The transformation was carried out using Agrobacterium tumefaciens GV3101 receptor cells. The fusion expression vector plasmid was introduced into *maize* protoplasts for subcellular localization, and the fluorescent signals were detected using a laser scanning microscope. The gene expression sites were identified through marker prediction and co-localization of the recombinant vectors.

### 4.4. Genetic Transformation of Arabidopsis thaliana

*Arabidopsis* homozygous mutant seeds containing T-DNA insertions were obtained from ABRC. These seeds underwent stress verification, and the subsequent generation was screened under varying Cd stress conditions. A basal medium of 1/2 MS solid medium, supplemented with Cd at concentrations of 0, 40, and 80 μmol/L, was used. Sixty seeds were inoculated per Petri dish, with three replicates per treatment. Root length and root hair development were then analyzed.

### 4.5. Validation of SpHMA3 Gene Overexpression in Arabidopsis thaliana

The *Arabidopsis* expression vector used was pRI101-AN, containing the 35S promoter, NOS terminator, KanR resistance marker gene, and BamHI digestion site. Following successful amplification, the *SpHMA3* gene cDNA was inserted into the overexpression vector through homologous recombination. The recombinant plasmid was first introduced into *E. coli* for plasmid extraction and then transferred to Agrobacterium. Wild-type *Arabidopsis thaliana* Columbia (Col) served as the material for transformation. The Agrobacterium containing the target gene was prepared into a transformation solution, in which the flowering *Arabidopsis thaliana* plants were submerged. The solution was covered with plastic film for 24 h before the film was removed for further cultivation. The seeds of the overexpressed materials were identified, and the T0 generation transgenic *Arabidopsis* seeds were germinated. DNA extracted from the leaves of *Arabidopsis* was further confirmed until no phenotypic segregation was observed. At this point, the transgenic *Arabidopsis* was confirmed to be purely positive and used in the subsequent experimental steps.

### 4.6. Arabidopsis Germination Phenotype Under Cd Stress

*Arabidopsis thaliana* T3 generation seeds, both positive and wild-type, were sown on 1/2 MS medium without KanR resistance. A total of 60 seeds were placed in each medium, with three replicates prepared. Following a 48 h vernalization period, the plates were incubated for 7 days. On the 7th day, the germination rate of the overexpressed lines and *WT* seeds was recorded. Additionally, the primary and total root lengths of *Arabidopsis thaliana* were measured on day 14.

### 4.7. Determination of Cd Content in Arabidopsis thaliana

*Arabidopsis OE* and *WT* were exposed to Cd stress at concentrations of 0, 40, and 80 µmol/L for 14 days. After treatment, the samples were fully dried to a constant weight, ground, and processed using a microwave ablation system (MARS 6) to prepare an ablation solution. The Cd content in the samples was then measured by an inductively coupled plasma mass spectrometer (NeXLON2000). Set the instrument to its optimum condition. The reference conditions of atomic absorption spectrophotometer are as follows: wavelength 228.8 nm, slit 0.2~1.0 nm, lamp current 2–10 mA, drying temperature 105 °C, drying time 20 s; Ashing temperature: 400~700 °C, ashing time: 20~40 s; The atomization temperature is 1300~2300 °C, and the atomization time is 3~5 s. Background correction was in the form of a deuterium lamp or the Zeeman effect. The LOD and LOQ for Cd were 0.001 mg/kg and 0.003 mg/kg, respectively.

### 4.8. Measurement of Physiological and Biochemical Indexes in Arabidopsis and Maize Seedlings

*Arabidopsis OE* and *WT* plants were exposed to 0, 40, and 80 µmol/LCd stress treatments. After 14 days of stress exposure, samples were collected to measure the levels of MDA, SOD, POD, and CAT in the plants, with the respective reaction systems detailed in Tables S6–S8. Additionally, roots of *KN5585* and SpHMA3 overexpression plants were harvested at 0, 12, 24, 36, 48, and 72 h under 800 μmol/L Cd treatment. Each time point was replicated three times, and MDA, SOD, POD, and CAT levels were assessed, with the corresponding systems shown in Tables S6–S8.

### 4.9. Overexpression of SpHMA3 Gene in Maize

The expression vector used was pCAMBIA3300, with primers incorporating enzyme restriction sites, and downstream primers containing terminators were designed. The method for vector construction followed the same procedure as that used for subcellular localization recombinant vectors, and the primer sequences are listed in Supplementary Table S9. Transgenic T0 plants were generated by infiltrating young *maize* embryos with Agrobacterium tumefaciens. DNA was extracted from T0 *maize* leaves, and positive identification was confirmed. The primers used for identification are provided in Table S10.

### 4.10. Determination of Cd Content in Various Parts of Maize

Overexpressed and *WT maize* seedlings were exposed to stress treatment using an 800 μmol/L CdCl_2_ solution for varying time points (0 h, 12 h, 24 h, 36 h, 48 h, and 72 h), and samples of their leaves and roots were collected to assess Cd accumulation in different plant tissues. Soil Cd concentrations were set at 0 mg/kg, 0.3 mg/kg, 3 mg/kg, 10 mg/kg, and 20 mg/kg in pots where both overexpression and *WT maize* were planted. Samples were collected at three growth stages: Maize-V6, Maize-VT, and Maize-R6, to measure the total Cd accumulation in the plants. Cd content was quantified by atomic absorption spectrophotometry, which involved preparing a Cd standard solution gradient, plotting a standard curve, and constructing a univariate linear regression equation to relate absorbance values to Cd concentration.

The Cd concentration in the sample was determined using the following equation:

(1) $$x=\frac{\left(C1-C0\right)\times V}{M\times 1000}$$

where plant enrichment factor (BCF) = Cplant/Csoil; plant transport factor (BTF) = Covergroundpart/Csubterraneanpart; *x*—Cd content in the specimen, in mg/kg^−1^; C1—Cd content in the specimen solution in ng/mL^−1^; C0—Cd content in the blank solution in ng/mL^−1^; V—the total volume of the specimen solution after calibration in mL. unit is mL; M—a mass of specimen in g; 1000—conversion factor; Cplant—heavy metal content in a tissue of a single plant (e.g., root, stem, leaf, male spike, female spike, seed); Csoil—concentration of the corresponding heavy metal elements in the soil (mg/kg^−1^); Covergroundpart—concentration of heavy metal elements in the above-ground part of the plant (mg/kg^−1^); Csubterraneanpart—concentration of a heavy metal element in the underground part of the plant (mg/kg^−1^).

### 4.11. Statistical Analysis

The data analysis was conducted with BM SPSS Statistics 26, GraphPad Prism 8, and Excel 2016, and the significant differences between the samples were evaluated by employing Student’s *t*-test.

## 5. Conclusions

This research explored *SpHMA3*, a key regulator of Zn and Cd uptake and translocation in the hyperaccumulator Sedum, which encodes a P-type ATPase family protein. Subcellular localization analysis demonstrated that *SpHMA3* protein resides in the *maize* cell membrane. The overexpression of *SpHMA3* significantly improved Cd tolerance in both *Arabidopsis thaliana* and *maize*, leading to an increased capacity for Cd uptake and translocation. These transgenic plants exhibited superior growth, enhanced antioxidant enzyme system activity, and greater accumulation of substances under Cd stress. The *AtHMA3* homozygous mutant displayed greater Cd sensitivity compared to *WT*. Analysis of Cd accumulation in overexpressed *maize* showed that more Cd was retained in the root system compared to the aerial parts, reducing the movement of Cd from soil to *maize* kernels and thereby mitigating the effects of Cd contamination. This work advances our understanding of the genetic and functional roles of *SpHMA3* and proposes a new, green, and efficient model for soil bioremediation of heavy metal Cd pollution while offering a valuable reference for *maize* in soil Cd pollution management.

## 6. Glossary

1. Heavy Metal ATPase (HMA): the HMA heavy metal transporter protein is a transmembrane transporter protein belonging to the P-type ATPase family, which mainly provides energy for metal transport by hydrolyzing ATP. This protein generally contains 6–8 transmembrane fragments and can selectively transport heavy metal cations, which plays an important role in phytoremediation of heavy metal contaminated soil.
2. Coding sequence (CDS): refers to a sequence that encodes a segment of a protein product. CDS is a structural genomics term that denotes the portion of a DNA sequence that is capable of being transcribed into mRNA and further translated into a protein.
3. Complementary DNA(cDNA): complementary (sometimes called copy) DNA, specifically a strand of DNA that is complementary to RNA after reverse transcription in vitro. Unlike what we normally call genomic DNA, cDNA does not have introns but only exon sequences.
4. Quantitative Real-time PCR(qRT-PCR): it is a method to measure the total amount of product after each polymerase chain reaction (PCR) cycle with fluorescent chemicals in a DNA amplification reaction. It is a method for quantitatively analyzing specific DNA sequences in the sample to be tested by internal or external reference methods.
5. Superoxide Dismutase(SOD): superoxide dismutase is also known as superoxide dismutase. It is a class of enzymes that catalyze the disproportionation of superoxide anion radicals (O_2_^−^) to H_2_O_2_ and O_2_. This enzyme is extremely widely distributed and has so far been isolated from a variety of organisms including bacteria, fungi, algae, plants, protozoa, insects, fish and mammals.
6. Peroxidase(POD): peroxidases are the hallmark enzymes of the peroxisome, its class of oxidoreductases, and they catalyze many reactions. Peroxidases are enzymes that catalyze the oxidation of substrates using hydrogen peroxide as an electron acceptor.
7. Catalase(CAT): it is an enzyme that catalyzes the breakdown of hydrogen peroxide into oxygen and water and is found in the peroxisomes of cells.
8. Malondialdehyde(MDA): it is an organic compound with the molecular formula C_3_H_4_O_2_, which belongs to the list of group 3 carcinogens.
9. Bioconcentration Factor (BCF): BCF is the ratio of the concentration of a compound in biological tissue (dry weight) to the concentration dissolved in water, or it can be considered as the ratio of the rate of uptake of the compound by the organism to the rate of purification of the compound from the organism, and it is used to indicate the magnitude of bioconcentration of organic compounds in the organism. The bioconcentration coefficient is an important indicator to describe the accumulation trend of chemical substances in organisms.
10. Plant Transport Coefficient(BTF): it is the ratio of the metal content in the above-ground part of the plant to the metal content in the below-ground part of the plant, and is used as an indicator to evaluate the ability of the plant to transport and enrich heavy metals from the below-ground part of the plant to the above-ground part of the plant.
11. Wild Type(*WT*): it is an individual or gene that has not been artificially mutated in nature. In genetics, a wild type is a phenotype that is present in more than 1% of a population and is often used as a standard control gene.
12. Thousand-grain Weight(TKW): it is the weight of 1000 grains of rice in grams. It is an indicator of the size and fullness of the seed, which is used to test the quality of the seed and the content of the crop test, and it is also an important basis for predicting yield in the field.

## Data Availability

The original data presented in the study are included in the article/Supplementary Materials. Further inquiries can be directed to the corresponding authors.

## Supplementary Materials

The following supporting information can be downloaded at: https://www.mdpi.com/article/10.3390/ijms26083487/s1.
