# Supplementary material for: SpHMA3: A Genetic Boost for Cadmium Tolerance and Bioremediation in Arabidopsis thaliana and Zea mays
Source: Int J Mol Sci. 2025 Apr 8;26(8):3487. doi: 10.3390/ijms26083487 (PMC12027283; doi:10.3390/ijms26083487)
Supplement: Supplementary file 1 [file ijms-26-03487-s001.zip › ijms-3395317-supplementary.pdf]

Schedules

Table S1 RT-PCR amplification primers

Gene name	Premier sequence Forward (5'---3')	Premier sequence Reverse (5'---3')
SpHMA3	ATGTGGATTTTTTTAGCC CTGCCTT	AAATAGTCCTTCAAAAAC ATCGTTC

Table S2 PCR dosing system

Ingredient	Volumetric(μ L)
Mix	7.5
Primer F	0.75
Primer R	0.75
cDNA	1.0
ddH2O	5.0
Total	15.0

Table S3 PCR reaction program

Temperature (° C)	Time
94	2 min
95	30 s
58	30 s
72	15 s
GO TO STEP2	34cycle
72	5 min
12	forever

Table S4 Semi-Quantitative RT-PCR amplification primers

Gene name	Premier sequence Forward(5’-3’)	Product size
GAPDH	F:ATCAACGGCTTCGGAAGGAT<br>R:CCGTGGACGGTGTCGTACTT	140bp
SpHMA3	F:TGGAGGCAAACGTAAGGAGC<br>R:GCCACAGCCACCCATTTTAG	120bp

Table S5 Semi-Quantitative RT-PCR dosing system

Ingredient	25 μL setup
2×T5 Super PCR Mix(Basic)	12.5 μL
10 μM upstream primer	1 μL
10 μM downstream primer	1 μL
cDNA	1 μL
ddH_2O	To 25 μL

Table S6 MDA detection system

Reagents（μL）	Survey	Blank
MDA assay working solution	300	300
Distilled water	-	100
Sample	100	-
Reagent III	100	100

Table S7 SOD enzyme activity determination system

Reagent（μL）	Survey	CK	Blank1	Blank2
Sample	18	18	-	-
Reagent I	45	45	45	45
Reagent II	2	-	2	-
Reagent III	35	35	35	35
Double distilled water	90	92	108	110
Reagent V	10	10	10	10

Table S8 POD enzyme activity determination system

Reagent（μL）	Survey	CK
Reagent I	45	45
Reagent II	2	-
Reagent III	35	35
Double-distilled water	90	92
Sample	10	10

Table S9 Construction of Overexpression Vector Primers with SpHMA3 Genes

Gene name	Premier sequence Forward (5'---3')	Premier sequence Reverse (5'---3')
SpHMA3	CGACTCTAGAGGATCCATGGATTCTGGATT GGATGA	CGGTACCCGGGGATCCACGTCTTTCAGGCA TCCATCT

Table S10 The primers were identified by over-expression of SpHMA3 in *maize*

Gene name	Premier sequence Forward (5'---3')	Premier sequence Reverse (5'---3')
SpHMA3	ATGTGGATTTTTTTAGCCCTGCCTT	AAATAGTCCTTCAAAAACATCGTTC

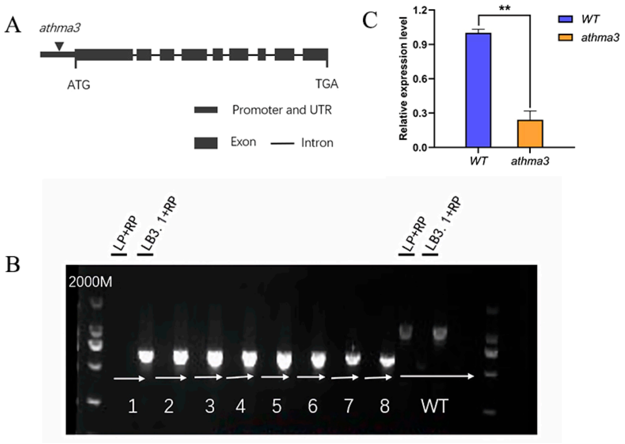

Supplementary figure S1. Analysis of the T-DNA insertion site in the *AtHMA3* gene of *Arabidopsis thaliana athma3* mutants, characterization of these mutants, and evaluation of *AtHMA3* transcript levels : (**A**) T-DNA insertion site analysis of *Arabidopsis thaliana athma3* mutants; (**B**) identification of *Arabidopsis thaliana athma3* mutants by the triple-primer method; and (**C**) analysis of transcript levels of the *AtHMA3* gene of *athma3* mutants under Cd stress. Note: (**C**), depicts the differential expression of the *AtHMA3* gene following 24 hours exposure to 80 μmol/L of Cd, * denotes $p \le 0.05$; ** denotes $p \le 0.01$; analyzed using Student's t-test.
